# Marked Increase in Anterior Ulnar Nerve Displacement at ≥90° of Elbow Flexion in Healthy Children

**DOI:** 10.2106/JBJS.OA.26.00115

**Published:** 2026-06-15

**Authors:** Tomoo Nakagawa, Youichi Yasui, Kenichi Kawabata, Jun Sasahara, Takahiro Inui, Masashi Nei, Yoichiro Konno, Hirotaka Kawano, Wataru Miyamoto

**Affiliations:** 1 Department of Orthopaedic Surgery, Teikyo University School of Medicine, Tokyo, Japan; 2 Trauma and Reconstruction Center, Teikyo University School of Medicine, Tokyo, Japan; 3 Department of Rehabilitation, Teikyo University Hospital, Tokyo, Japan

## Abstract

**Background:**

Iatrogenic ulnar nerve (UN) injury is a serious complication of percutaneous medial pinning for pediatric supracondylar humeral fractures, particularly when deep elbow flexion is required for reduction. However, the flexion angle at which the nerve becomes unstable and patient characteristics predisposing to displacement remain unclear. This study aimed to determine the elbow flexion angle at which UN instability occurs in healthy children and to identify independent risk factors for anterior displacement.

**Methods:**

A cross-sectional study of 74 healthy, asymptomatic children (144 elbows) aged 7 to 12 years was conducted. Dynamic ultrasonography was performed at 5 static flexion angles (0°, 30°, 60°, 90°, and 120°), with nerve position classified as normal, subluxated, or dislocated relative to the medial epicondyle. Multivariate logistic regression analysis identified independent predictors of instability (subluxation or dislocation) at deep flexion (≥90°), evaluating age, sex, body mass index, handedness, and generalized joint laxity.

**Results:**

UN instability increased progressively with elbow flexion. At 0° and 30°, the nerve remained stable in >97% of elbows. A marked increase in instability was observed at ≥90°: 60.4% demonstrated instability (50.0% subluxation, 10.4% dislocation) at 90°, rising to 94.4% (54.2% subluxation, 40.3% dislocation) at 120°. Multivariate analysis identified female sex (odds ratio 3.61; 95% confidence interval 1.50-8.67) and generalized joint laxity (odds ratio 2.61; 95% confidence interval, 1.03-6.61) as independent risk factors. Age, body mass index, and handedness were not significantly associated with displacement.

**Conclusions:**

In healthy children, the UN demonstrated a pronounced angle-dependent pattern, with marked anterior displacement at ≥90° of flexion. Female sex and generalized joint laxity were associated with higher odds of sonographic instability. These findings characterize the anatomical behavior of the UN during elbow flexion and provide context for future investigation in surgical populations.

**Level of Evidence:**

Level III. See Instructions for Authors for a complete description of levels of evidence.

## Introduction

Iatrogenic ulnar nerve (UN) injury remains a serious complication of medial pinning for pediatric supracondylar humerus fractures^1-3^. The risk increases significantly with deep elbow flexion for fracture reduction, narrowing the cubital tunnel and displacing the UN anteriorly into the trajectory of percutaneous K-wires^3-8^. Reported injury rates vary, with incidences of up to 20%^2,3,9-11^. Even transient UN palsy may result in persistent deficits, underscoring the need for preventive strategies supported by robust biomechanical evidence^4,5^.

Prior investigations using ultrasonography and magnetic resonance imaging have demonstrated that elbow flexion increases intraneural pressure and may displace the UN anteriorly, with effects potentially accentuated in children by skeletal immaturity and ligamentous laxity^5,7,12-15^. However, 2 critical gaps remain. First, previous ultrasonographic studies have used heterogeneous definitions of subluxation and dislocation and have often lacked systematic, angle-specific assessments, leaving displacement probability at incremental flexion angles poorly defined. Second, patient-level predictors of anterior displacement remain controversial^4-8^. Consequently, surgeons lack precise evidence to anticipate the UN location at specific intraoperative flexion angles during medial pinning.

This study aimed to (1) determine the flexion angles at which the UN demonstrates anterior displacement in healthy children using dynamic ultrasonography and (2) identify independent risk factors for such displacement.

## Material and Methods

### Study Design and Participants

This cross-sectional observational study received approval from the Institutional Review Board of the affiliated university hospital (IRB No. 24-0103) and was conducted in accordance with the Declaration of Helsinki and Strengthening the Reporting of Observational Studies in Epidemiology guidelines. Race and ethnicity data were not collected.

Asymptomatic Japanese elementary school students were recruited from a local youth soccer club between April 2024 and March 2025. Exclusion criteria included previous elbow fracture, acute injury during examination, and investigator-determined unsuitability. Written informed consent and assent were obtained from legal guardians and participants, respectively.

### Ultrasound Examination

**Fig. 1:**
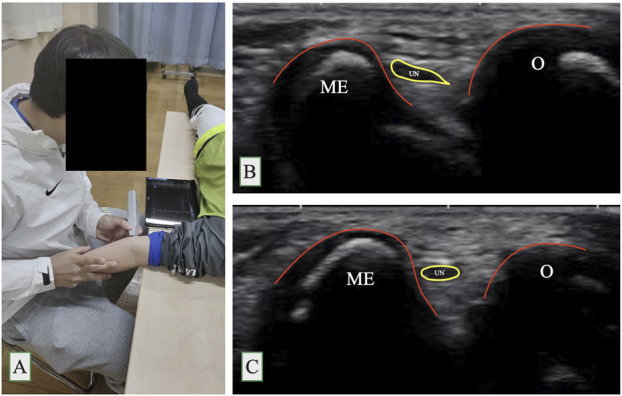
Standardized positioning and transducer protocol for dynamic ultrasonographic evaluation of the UN. **Fig. 1-A** A board-certified orthopedic surgeon performing dynamic ultrasonography on a child in the standardized supine position with the shoulder abducted and elbow flexed according to the study protocol. **Fig. 1-B** The transducer is placed transversely between the ME and olecranon to obtain a short-axis view of the UN within the cubital tunnel. **Fig. 1-C** The probe is slid to visualize the apex of the ME and then tilted until the ultrasound beam is perpendicular to the UN, obtaining an optimized short-axis image with circular or elliptical morphology. ME = medial epicondyle, O = olecranon, and UN = ulnar nerve.

All ultrasonographic assessments were performed by 1 of 2 board-certified orthopedic surgeons, each with >10 years of experience in musculoskeletal ultrasound, using a high-frequency linear transducer, and 2 ultrasound machines (SONIMAGE MX1, Konica Minolta; Viewphii-US, Socionext). Participants were positioned supine with the shoulder abducted to 90° and the forearm neutral, simulating the standard intraoperative setup for supracondylar humerus fracture fixation (Fig. 1A)^16^. To standardize transducer placement, the probe was first aligned along the medial epicondyle (ME)-olecranon axis in the transverse plane (Fig. 1B), then repositioned perpendicular to the skin surface at the apex of the ME to obtain a cross-sectional view of the UN (Fig. 1C).

The elbow was passively moved through a sequence of 5 static flexion angles: 0°, 30°, 60°, 90°, and 120°. The angle was measured using a standard goniometer.

### UN Positional Classification

**Fig. 2:**
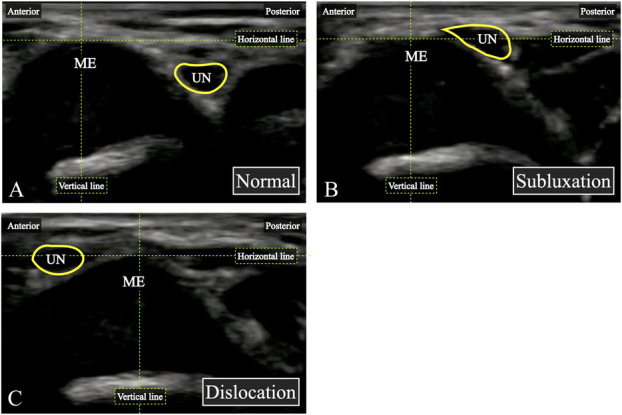
UN positional classification on short-axis ultrasound. The position of the UN was classified relative to 2 reference lines drawn from the apex of the ME. **Fig. 2-A** Type N (normal): The UN is located posterior to the vertical reference line and deep to the horizontal reference line within the cubital tunnel. **Fig. 2-B** Type S (subluxation): The UN is located posterior to the vertical reference line but superficial to the horizontal reference line. **Fig. 2-C** Type D (dislocation): The UN is located anterior to the vertical reference line. ME = medial epicondyle, and UN = ulnar nerve.

The position of the UN was classified relative to 2 orthogonal reference lines drawn from the apex of the ME. A vertical line was drawn perpendicular to the skin surface through the apex of the epicondyle, and a horizontal line was drawn parallel to the skin surface through the same apex^17,18^. Based on these lines, the nerve position was categorized into 1 of 3 types, adapted from previous literature^17,18^ (Fig. 2).

**Type N (normal)**: The UN is located posterior to the vertical line and deep to the horizontal line within the cubital tunnel.

**Type S (subluxation)**: The UN is located posterior to the vertical reference line but superficial to the horizontal reference line.

**Type D (dislocation)**: The UN is located anterior to the vertical reference line.

A strict threshold was applied to ensure sensitive detection of anterior nerve translation. Nerve crossing of the horizontal or vertical reference line was classified as Type S or Type D, respectively. This classification reflects the clinical relevance of anterior translation: medial pin insertion is typically performed at the apex of the ME, and nerve displacement toward or beyond this landmark may place it directly in the Kirschner wire trajectory.

### Image Analysis and Clinical Data Collection

All images were anonymized and independently reviewed by 2 orthopedic surgeons (the same surgeons who performed the examinations). The reviewers were blinded to the participants’ clinical information and the flexion angle at which the images were obtained to minimize potential observer bias. A third senior pediatric orthopedic surgeon reviewed classifications with disagreement, and the final classification was determined by consensus. Data on age, sex, dominant hand, and body mass index (BMI) were collected for each participant. Generalized joint laxity was assessed using the Wynne-Davies criteria, and was defined as a score of ≥3 out of 5^19^.

### Reliability Analysis

Two independent raters evaluated a randomized subset of 150 images (30 elbows × 5 angles). Linearly weighted Cohen's κ was used given the ordinal classification; intra-rater reliability was assessed after a 2-week washout period^20,21^. Agreement was interpreted per Soleimani et al., with raw percent agreement reported where κ was undefined due to zero variance^22^.

### Statistical Analysis

The primary outcome was the distribution of the UN positional types (Normal, Subluxated, Dislocated) at the 5 elbow flexion angles. The proportional odds ratio [OR] assumption was verified using Brant’s test.

Risk factors associated with UN instability (Type S or D) in deep flexion (≥90°) were evaluated as a secondary outcome using multivariable logistic regression with age, sex, side (dominant vs. nondominant), BMI, and generalized joint laxity. McNemar’s test was used to compare the proportion of unstable nerves between low flexion (≤60°) and high flexion (≥90°) within participants.

Sample size was determined a priori to detect a significant shift in nerve instability using McNemar's test. Although previous studies reported instability rates of approximately 40% in flexion, an absolute 20% increase was estimated in unstable nerves from low (≤60°) to high (≥90°) flexion to ensure adequate statistical power^4,8,23^. A minimum of 130 elbows was required to achieve 80% power with a two-sided alpha level of 0.05. All statistical analyses were performed using STATA version 16.0 (StataCorp LLC, College Station, TX, USA), with statistical significance set at p < 0.05.

## Results

### Participant Characteristics

Seventy-four healthy children (59 boys, 144 elbows) were enrolled. Participant demographics are presented in Table I. No adverse events occurred, and no data were missing for outcome.

**TABLE I:** Participant Demographics and Characteristics

Characteristic	Value
No. of participants	74
Sex (% male)	79.7
Mean age (range) (yr)	9.7 (7-12)
Mean BMI (SD)	16.4 (1.93)
No. of handedness (%)	
Right-handed	64 (86.5)
Left-handed	9 (12.2)
Ambidextrous	1 (1.3)
Generalized joint laxity	
No. of Wynne-Davies score ≥3, n (%)	25 (33.8)
Median Score (IQR)	2 (1-3)

BMI = body mass index, IQR = interquartile range.

### Primary Outcome: UN Position Across Flexion Angles

The distribution of UN positions at each flexion angle is summarized in Table II. At 0°, the nerve was normal in all but 1 elbow (0.7% subluxation), with displacement increasing progressively with flexion; at 120°, more than half demonstrated displacement beyond the ME. Ordinal logistic regression confirmed a significant association between flexion angle and nerve displacement (p < 0.001), with an OR of 1.07 per 1° increase (95% confidence interval [CI], 1.06-1.08; equivalent to OR 8.34 per 30°; 95% CI, 6.31-11.03). The proportional odds assumption was not violated (Brant test, p = 0.21).

**TABLE II:** Ulnar Nerve Position by Elbow Flexion Angle

Flexion Angle	Type N, n (%)	Type S, n (%)	Type D, n (%)
0°	143 (99.3)	1 (0.7)	0 (0.0)
30°	140 (97.2)	4 (2.8)	0 (0.0)
60°	126 (87.5)	17 (11.8)	1 (0.7)
90°	57 (39.6)	72 (50.0)	15 (10.4)
120°	8 (5.5)	78 (54.2)	58 (40.3)

D = dislocation, N = Normal, and S = subluxation.

McNemar's test demonstrated a significant increase in instability beyond 90°: of 126 elbows classified as stable at ≤60°, 118 (93.7%) developed new-onset subluxation or dislocation at ≥90° (matched-pair OR 237; 95% CI, 15-3,800; p < 0.001).

### Secondary Outcome: Risk Factors for Instability at ≥90°

Multivariate logistic regression identified female sex (OR 3.61; 95% CI, 1.50-8.67; p = 0.004) and generalized joint laxity (OR 2.61; 95% CI, 1.03-6.61; p = 0.043) as independent risk factors for UN instability at ≥90°. Age, BMI, and handedness were not significant (Table III). Multicollinearity was absent (maximum variance inflation factor = 2.23), and the model demonstrated acceptable fit (pseudo R^2^ = 0.094; likelihood ratio χ^2^(6) = 18.22; p = 0.006).

**TABLE III:** Multivariate Logistic Regression Analysis of Risk Factors for Ulnar Nerve Instability at ≥90° Elbow Flexion

Variable	OR	95% CI	p
Generalized joint laxity	2.61	1.03-6.61	0.043
Female sex	3.61	1.50-8.67	0.004
Age	0.89	0.71-1.12	0.321
BMI	1.06	0.88-1.28	0.520
Dominant hand (nondominant)	1.60	0.68-3.79	0.281

BMI = body mass index, CI = confidence interval, and OR = odds ratio.

Among 70 participants with bilateral data, concordant nerve classification at ≥90° was observed in 77.1% of pairs (McNemar's test, p = 0.012).

### Reliability

Inter-rater agreement was almost perfect (weighted κ 0.88; 95% CI, 0.81-0.94), with intra-rater reliability similarly high for both raters (κ 0.95 and 0.90). Angle-specific agreement ranged from substantial to almost perfect (κ 0.86-1.00), except at 0°, where universal “normal” classification yielded 100% raw agreement (κ undefined).

## Discussion

This study characterizes the angle-dependent behavior of the UN in healthy children, with a marked increase in anterior displacement at ≥90° of flexion—a position frequently required for fracture reduction during medial pinning of supracondylar humerus fractures, where iatrogenic injury risk is highest^4,9,24^. Furthermore, female sex and generalized joint laxity were identified as independent predictors of displacement, providing evidence for patient-specific susceptibility.

These findings bridge the critical gap between anatomical understanding and intraoperative uncertainty in fracture fixation. In a sonographic study of 466 asymptomatic children under 14 years, Gao et al. reported that UN instability increased with elbow flexion, although their assessment relied on continuous motion without discrete angle-specific evaluation^6^. Shen et al. examined 237 healthy children aged 6 to 11 years at 0°, 45°, 90°, and 120°, observing nerve flattening and a tendency for dislocation at higher flexion angles^5^. They did not report precise instability rates at each angle, and the positional definitions of subluxation versus dislocation remained unclear^5^. This study addressed these limitations through a standardized five-angle protocol and rigorous statistical analysis. Anterior displacement increased sharply at ≥90° of flexion, affecting 60% of elbows at 90° and over 90% at 120°, underscoring the strong angle dependence of UN instability.

While previous studies in healthy adults and children have reported rates of 37% to 58%, this study observed instability in over 90% of elbows^5,8,17,23^. This likely reflects not overestimation but deliberate replication of the deep elbow flexion angles required during medial pinning^24,25^. Whereas earlier studies assessed the nerve with the shoulder in mild abduction during seated or standing evaluations, this study employed a supine position with 90° shoulder abduction, closely mirroring operative conditions^2,5,8,17,23,25^. These findings suggest that under clinically relevant conditions, the UN reliably resides near the ME.

Although age, generalized joint laxity, and repetitive upper extremity use have previously been implicated in UN instability, the literature remains inconsistent, particularly regarding age-related risk profiles^6,8,18,23^. The association with generalized joint laxity suggests that constitutional ligamentous laxity compromises soft-tissue stabilizers of the cubital tunnel, including the Osborne ligament, facilitating anterior nerve translation^26^. The independent effect of female sex may reflect developmental or morphological characteristics such as differences in carrying angle or elbow hyperextension, though these were not directly assessed^26,27^. Collectively, female sex and generalized joint laxity—both reflecting underlying ligamentous or morphological vulnerability—were associated with increased odds of sonographic displacement in this cohort.

While deep flexion displaces the UN, this instability does not directly equate to iatrogenic UN injury during medial pinning of supracondylar humerus fractures. This discrepancy may reflect high nerve mobility, cautious pin trajectories, and underrecognized subclinical neuropraxia^3,26,28^. Moreover, because supracondylar fractures most commonly occur in children younger than those studied here, these findings may represent a conservative estimate of risk in the typical fracture population^29^. Importantly, the distinction between Type S and Type D carries distinct surgical implications. In Type S, the nerve rests at the ME apex, potentially within the trajectory of medially inserted Kirschner wires. In Type D, anterior displacement may place the nerve in a position less readily appreciated during pin insertion; furthermore, subsequent elbow extension may bring it into closer proximity to the wire.

From a clinical standpoint, our findings underscore the need for strategic caution during medial pinning in pediatric supracondylar humerus fractures. Fracture reduction often requires deep elbow flexion—precisely the position in which the UN is most frequently displaced anteriorly toward the medial pin corridor^29,30^. The standardized ultrasonographic protocol described in this study can be implemented both preoperatively for risk stratification and intraoperatively for real-time nerve localization. This technique enables rapid, reproducible assessment without disrupting workflow. In high-risk patients (women with generalized joint laxity) or when anterior displacement is detected, surgeons may consider mini-open techniques, modified pin trajectories, or reduced flexion angles if fracture stability permits^1,4,25,28,31^. The identification of displacement even at 0° of flexion, although rare, further suggests that preoperative assessment may provide safety assurance regardless of elbow position. Moreover, bilateral comparison in 70 participants showed only 77% concordance (p = 0.012), suggesting that the contralateral elbow cannot reliably substitute for direct assessment. Together, these observations highlight the importance of patient-specific anatomical assessment in surgical planning.

This study had several limitations. First, ultrasonographic interpretation is inherently subjective, although this was mitigated by standardized protocols, blinded independent evaluation, and adjudication by a senior pediatric orthopedic surgeon; weighted κ values were 0.88 (inter-rater) and 0.90 to 0.95 (intra-rater), indicating almost perfect agreement^22^. Observer bias related to image familiarity cannot be entirely excluded. Second, the use of 2 ultrasound systems may have introduced minor variability in image quality. Third, participants were healthy youth soccer players from a single geographic region, predominantly male; as soccer imposes minimal upper-extremity loading, activity-related adaptations are unlikely, and the mean BMI was consistent with national age-specific averages^32^. Nevertheless, generalizability to populations of different sex distributions, ethnicities, or socioeconomic backgrounds may be limited. Fourth, bilateral elbows were treated as independent observations. Finally, this study evaluated only uninjured elbows; how fracture-related swelling, hematoma, or soft-tissue injury affects UN stability remains uncertain, and this warrants particular consideration when interpreting these findings in supracondylar humerus fractures.

## Conclusion

This cross-sectional study demonstrated that UN displacement increases substantially at flexion angles of 90° and above in healthy children, with a high prevalence of anterior translation during deep elbow flexion. Female sex and generalized joint laxity were associated with higher odds of sonographic instability in this cohort.

## Funding

This study was supported by a research grant from the Japan Orthopaedics and Traumatology Foundation (Grant No. 475, fiscal year 2020). The funding source had no role in the design, conduct, analysis, interpretation, or preparation of the manuscript.

## Data Availability

The data sets generated and analyzed during this study are available from the corresponding author on reasonable request.
